# An atlas of glucose uptake across the entire human body as measured by the total-body PET/CT scanner: a pilot study

**DOI:** 10.1093/lifemeta/loac030

**Published:** 2022-10-27

**Authors:** Weizhao Lu, Zhaoping Cheng, Xue Xie, Kun Li, Yanhua Duan, Min Li, Chao Ma, Sijin Liu, Jianfeng Qiu

**Affiliations:** Department of Radiology, Shandong First Medical University & Shandong Academy of Medical Sciences, Taian, Shandong 271016, China; Department of PET/CT, the First Affiliated Hospital of Shandong First Medical University, Shandong Provincial Qianfoshan Hospital Affiliated to Shandong University, Jinan, Shandong 250014, China; Department of Radiology, Shandong First Medical University & Shandong Academy of Medical Sciences, Taian, Shandong 271016, China; Department of PET/CT, the First Affiliated Hospital of Shandong First Medical University, Shandong Provincial Qianfoshan Hospital Affiliated to Shandong University, Jinan, Shandong 250014, China; Department of PET/CT, the First Affiliated Hospital of Shandong First Medical University, Shandong Provincial Qianfoshan Hospital Affiliated to Shandong University, Jinan, Shandong 250014, China; Department of Radiology, Shandong First Medical University & Shandong Academy of Medical Sciences, Taian, Shandong 271016, China; Department of Radiology, Shandong First Medical University & Shandong Academy of Medical Sciences, Taian, Shandong 271016, China; Science and Technology Innovation Center, Shandong First Medical University & Shandong Academy of Medical Sciences, Jinan, Shandong 250100, China; State Key Laboratory of Environment Chemistry and Ecotoxicology, Research Center for Eco-Environment Sciences, Chinese Academy of Sciences, Beijing 100085, China; Department of Radiology, Shandong First Medical University & Shandong Academy of Medical Sciences, Taian, Shandong 271016, China

**Keywords:** glucose uptake, PET/CT, glucose uptake atlas, uEXPLORER

## Abstract

Glucose uptake differs in organs and tissues across the human body. To date, however, there has been no single atlas providing detailed glucose uptake profiles across the entire human body. Therefore, we aimed to generate a detailed profile of glucose uptake across the entire human body using the uEXPLORER positron emission tomography/computed tomography scanner, which offers the opportunity to collect glucose metabolic imaging quickly and simultaneously in all sites of the body. The standardized uptake value normalized by lean body mass (SUL) of 18F-fluorodeoxyglucose was used as a measure of glucose uptake. We developed a fingerprint of glucose uptake reflecting the mean SULs of major organs and parts across the entire human body in 15 healthy-weight and 18 overweight subjects. Using the segmentation of organs and body parts from the atlas, we uncovered the significant impacts of age, sex, and obesity on glucose uptake in organs and parts across the entire body. A difference was recognized between the right and left side of the body. Overall, we generated a total-body glucose uptake atlas that could be used as the reference for the diagnosis and evaluation of disordered states involving dysregulated glucose metabolism.

## Introduction

Metabolism, whether at the level of small molecules, protein homeostasis, signaling patterns, or inter-tissue communication, underpins several vital processes that ultimately sustain life through a set of chemical reactions in organisms [1]. Since glucose is the principal energy source for most cells, many organisms have evolved numerous and sophisticated mechanisms to sense glucose and respond to it appropriately [2]. In contrast to the situation in unicellular organisms, such as bacteria or yeasts, human metabolism is an integral part of cellular function, relying on multiple cells to work in concert to achieve metabolic homeostasis [3]. This characteristic, in turn, allows organs to develop specialized metabolism, i.e. adapted to their particular biological functions [4]. Therefore, it is of biological significance to understand the glucose metabolism of human organs in a holistic manner. Assessing metabolism at the system level is also useful in exploring the origin and progression of various diseased conditions, including obesity, diabetes, hypertension, neurodegenerative diseases, and cardiovascular diseases, since these conditions are associated with abnormal metabolic states [5]. In addition, normal total-body metabolism at the system level can be used as the reference in evaluating the efficacy of therapeutics against diverse diseases, including cancers and metabolic syndrome [6].

Total-body glucose metabolism has been evaluated in several animal models, such as rats and pigs [4, 7–9]. Nonetheless, there has been a few system-level analysis of glucose metabolism in humans. In the past several decades, metabolic imaging has developed rapidly, facilitating large-scale studies that have highlighted the importance of metabolic dynamics in a wide range of diseases, including cancers, diabetes, and cardiovascular diseases [10]. Positron emission tomography (PET) is a powerful tool for the visualization of metabolism, especially the *in vivo* measurement of glucose uptake, which is an important step of glucose metabolism [11, 12]. Recent studies have assessed glucose uptake in the brain, visceral adipose tissue, and liver using PET imaging [13–15]. However, due to the inadequate axial field of view (FOV) of the existing PET scanners, simultaneous measurement of glucose uptake in major organs and parts across the entire human body has not yet been achieved.

With the recent advent of the total-body PET/computed tomography (CT) scanner by the EXPLORER consortium, it was hypothesized that the detailed glucose uptake profiles across the human body could be generated with the aid of the total-body PET/CT. Additionally, previous studies have reported that age, sex, and obesity had effects on total-body glucose uptake, because emerging evidence indicates that metabolic alterations accumulate over time during the human aging process [16–18], and sexual dimorphism introduces alterations in the metabolic profiles of several organs distributed throughout the human body [19, 20]. Therefore, in this study, the total-body uEXPLORER PET/CT scanner was applied to develop a human glucose uptake atlas encompassing major organs and body parts throughout the body and reflecting the regional variation of glucose uptake in the brain. In addition, this atlas was used to investigate the effect of age, sex, lateralization, and obesity on glucose uptake, in order to prove its role in understanding glucose metabolism across the human body.

## Results

**Figure 1:**
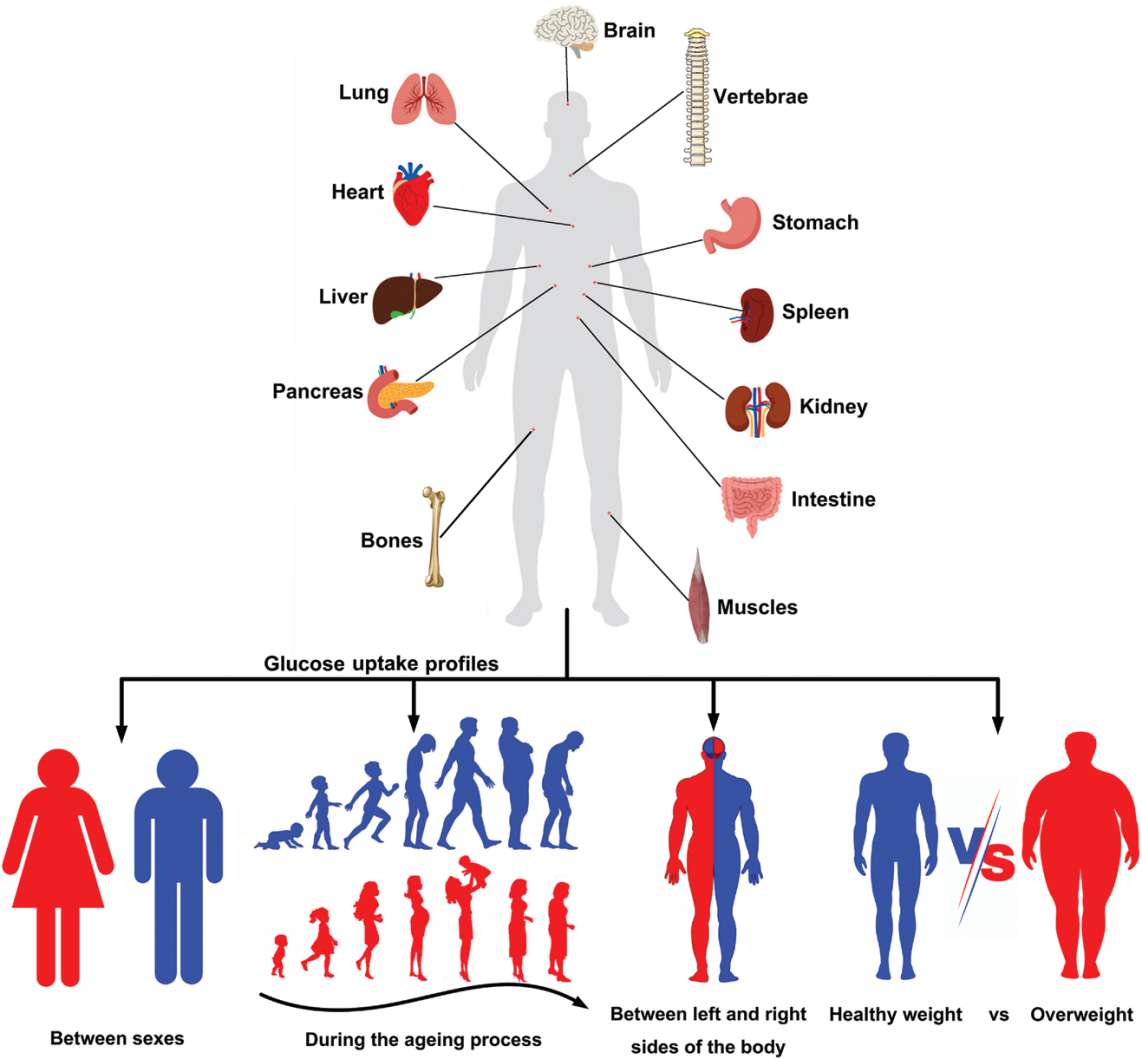
A schematic diagram showing the detection of glucose uptake activity. Total-body glucose uptake differences are assessed between sexes, between the left and right sides of the body (each side of the brain hemisphere corresponds to the contralateral body part), among different age groups, and between healthy-weight and overweight subjects.

To develop a general atlas of glucose uptake activity across the whole body among the healthy-weight subjects, we calculated the standardized uptake values (SUVs) normalized by lean body mass (SUL) of the major organs and body parts, and compared the values between sexes, sides, and age groups (Fig. 1). In addition, we compared the SULs of major organs and body parts between the healthy-weight and overweight groups (Fig. 1). Associations among different parameters were also analyzed.

### Glucose uptake atlas of the human body

**Figure 2:**
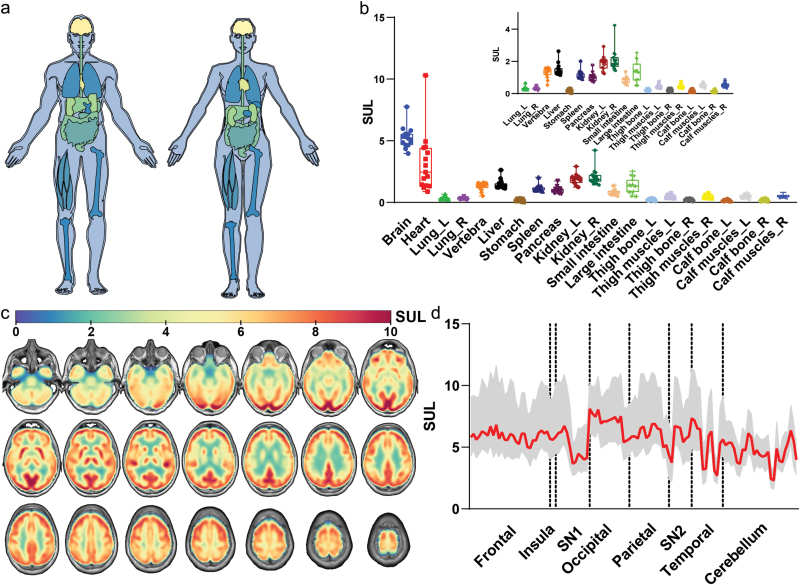
Glucose uptake activity in organs and parts throughout the human body in healthy-weight subjects. (a) A representative atlas of glucose uptake calculated by the mean SULs of 18F-FDG in major organs and parts throughout the body. The SULs in different organs and parts were averaged across the 15 healthy-weight subjects. (b) Box plot of the mean SULs in organs and parts throughout the body for each subject. L: left; R: right. The error bar indicates mean ± standard deviation. (c) Zonation of glucose uptake in the brain, shown in axial slices ordered from inferior to superior. The SUL maps are averaged across the 15 healthy-weight subjects. (d) Glucose uptake among different brain regions. The solid line represents the mean SUL across the 15 healthy-weight subjects. The filled area represents the standard deviation. SN: subcortical nuclei. SN1 includes the cingulate gyrus, hippocampus, parahippocampal gyrus, and amygdala. SN2 includes the putamen, pallidum, and thalamus.

Fig. 2 shows the glucose uptake of major organs and parts throughout the human body, calculated according to the mean SUL (the raw PET images for the 15 healthy subjects are displayed in Supplementary Fig. S1). Among the major organs and body parts, the brain exhibited the highest glucose uptake value, while the bilateral lungs, bones, and muscles manifested the lowest glucose uptake activity (Fig. 2a and b). Other metabolizing organs such as the heart and liver also had relatively high glucose uptake (Fig. 2b).

Within the brain, the occipital lobe manifested the greatest glucose uptake, but several brain regions, including subcortical nuclei, the temporal lobe, and the cerebellum, may be deactivated in the resting state, resulting in decreased activity and fluctuations in glucose uptake (Fig. 2c and d).

### The role of age in determining glucose uptake

**Figure 3:**
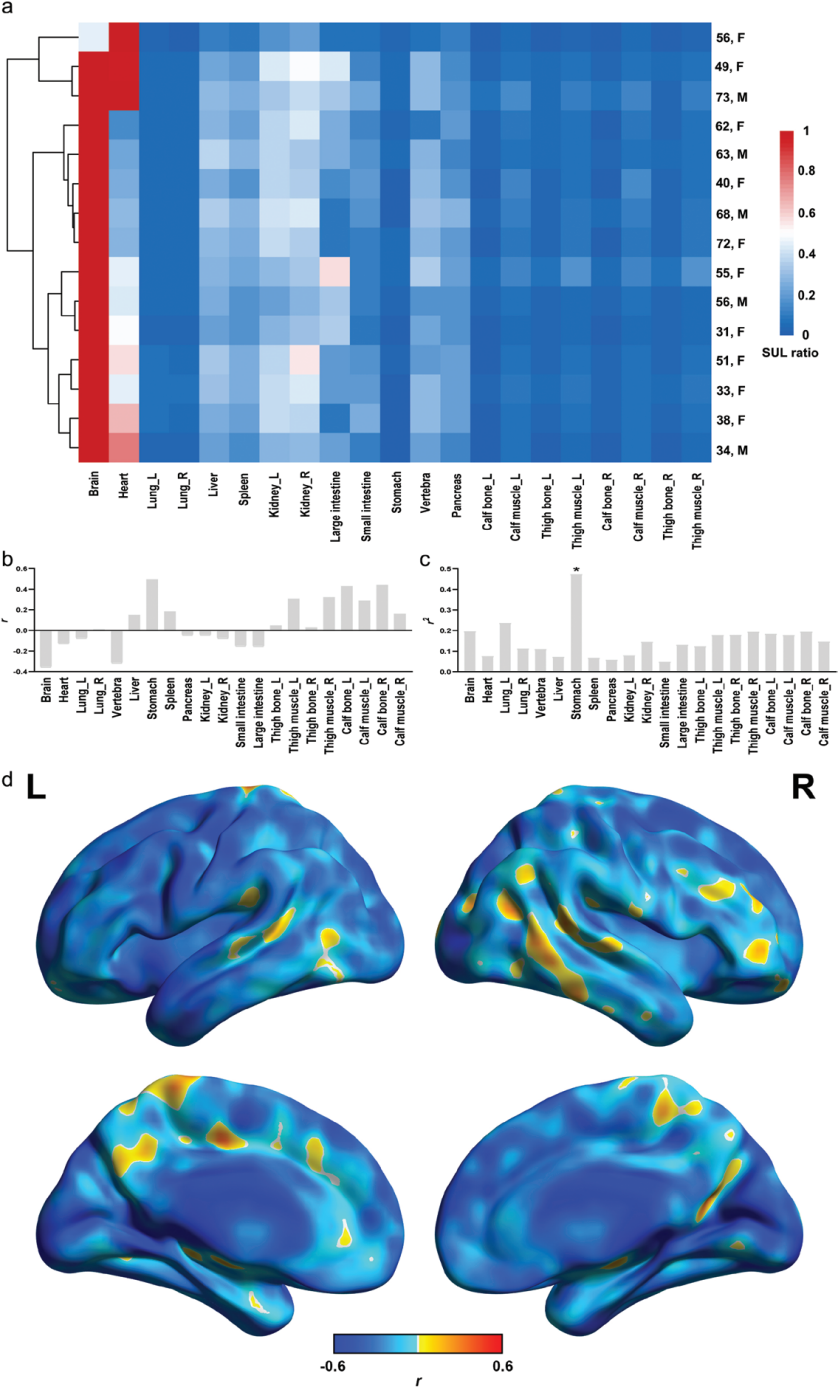
Association between glucose uptake and age in major organs and parts across the entire body. (a) Heatmap of SUL ratio (divided by the maximum value) in major organs and body parts of the 15 healthy-weight subjects (the raw PET images for the 15 healthy subjects are displayed in Supplementary Fig. S1). Each row represents a single subject, and each column represents the SUL ratio of an organ or body part. Samples are clustered using hierarchical clustering. (b) Correlation between SUL in major organs or parts across the entire body and age calculated by Pearson correlation analysis. (c) Association between SUL in major organs or body parts and age calculated by quadratic regression analysis. (d) Correlation maps between brain SUL and age shown in brain render maps.

To understand the influence of age on glucose uptake, we investigated the association between age and SUL of organs and parts across the human body (Fig. 3). Notably, clustering of the healthy-weight subjects based on the SUL ratio (min–max scaling) revealed that age had a large effect on glucose uptake, as people in their 30s, and people in their 60s or older formed separate clusters when grouped by the mean SUL of organs and parts throughout the body (Fig. 3a). Visualization and results of the association between age and SULs in other organs or body parts are shown in Supplementary Fig. S2 and Supplementary Table S1.

Correlation and regression analysis revealed that the SULs in several organs, including the brain, vertebra, thigh muscle, calf bone, and calf muscle, etc., showed associations with age (Fig. 3b, Supplementary Fig. S2 and Supplementary Table S1). SUL of the stomach had significant inverted U-shape associations with age (Fig. 3c and Supplementary Fig. S2). In terms of brain SUL map, negative associations with age were observed across the entire brain (Fig. 3d), and significant negative associations with age were observed in the corpus callosum (Supplementary Fig. S3 and Supplementary Table S2).

### The role of sex in determining glucose uptake

**Figure 4:**
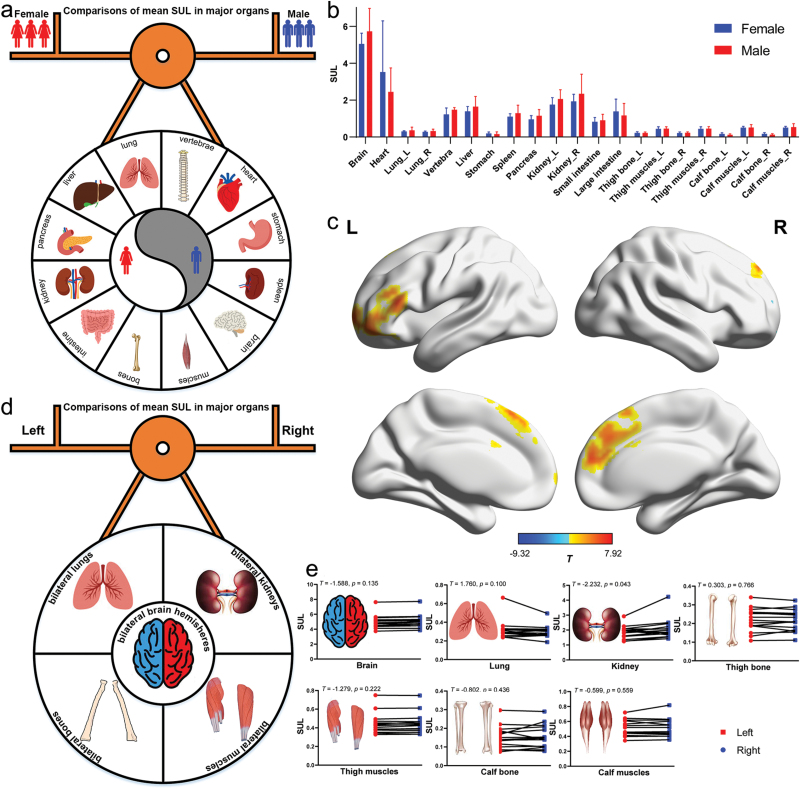
Differences in glucose uptake of major organs and parts of the human body caused by sex and by laterality (left or right) in the healthy-weight group. (a) Overview of the comparisons of the mean SULs of major organs and body parts between sexes. (b) Comparisons of SULs in major organs and parts between male and female subjects, expressed as mean ± standard deviation. (c) Comparisons of SUL ratio maps of the brain between male and female subjects (Gaussian corrected at voxel level *P* < 0.01, cluster level *P* < 0.05). The corresponding permutation maps are shown in Supplementary Fig. S4. (d) Overview of comparisons of the mean SULs of major organs and parts between the left and right sides of the body. (e) Comparisons of the mean SULs of brain hemispheres, lungs, kidneys, bones, and muscles between the left and right sides of the body.

In this study, we compared the mean SULs of major organs and body parts between sexes (Fig. 4a), and the results are demonstrated in Fig. 4b and c. Fig. 4b indicates no significant differences in the mean SULs of major organs and body parts between male and female subjects. Sexual dimorphism was observed in the frontal lobe of the brain (Fig. 4c, Supplementary Table S3 and Supplementary Fig. S4).

### The role of lateralization in determining glucose uptake

In order to explore the role of lateralization in determining glucose uptake, paired *t*-tests were performed to compare the mean SULs of major organs and parts between the right and left sides of the body, such as the bilateral brain hemispheres, bilateral lungs, bilateral kidneys, bilateral bones, and bilateral muscles (Fig. 4d). The results showed that lateralized differences in mean SULs existed in the kidney, while no lateralized differences were found in other organs across the body (Fig. 4e).

Within the brain, several brain regions including the middle frontal gyrus, orbital part of middle frontal gyrus, opercular part of inferior frontal gyrus, middle cingulate gyrus, posterior cingulate gyrus, cuneus, inferior occipital gyrus, caudate nucleus, middle temporal pole, pallidum, crus 2 of the cerebellum, cerebellum 7b showed laterality in the mean SULs between the left and right structures (Supplementary Fig. S5 and Supplementary Table S4). Specifically, the left posterior cingulate gyrus, caudate nucleus, pallidum, middle temporal pole, and crus 2 of the cerebellum displayed higher glucose uptake than their contralateral parts. In contrast, the remaining left regions showed lower glucose uptake than their counterparts (Supplementary Fig. S5).

### Differences in glucose uptake between the healthy-weight and the overweight groups

**Figure 5:**
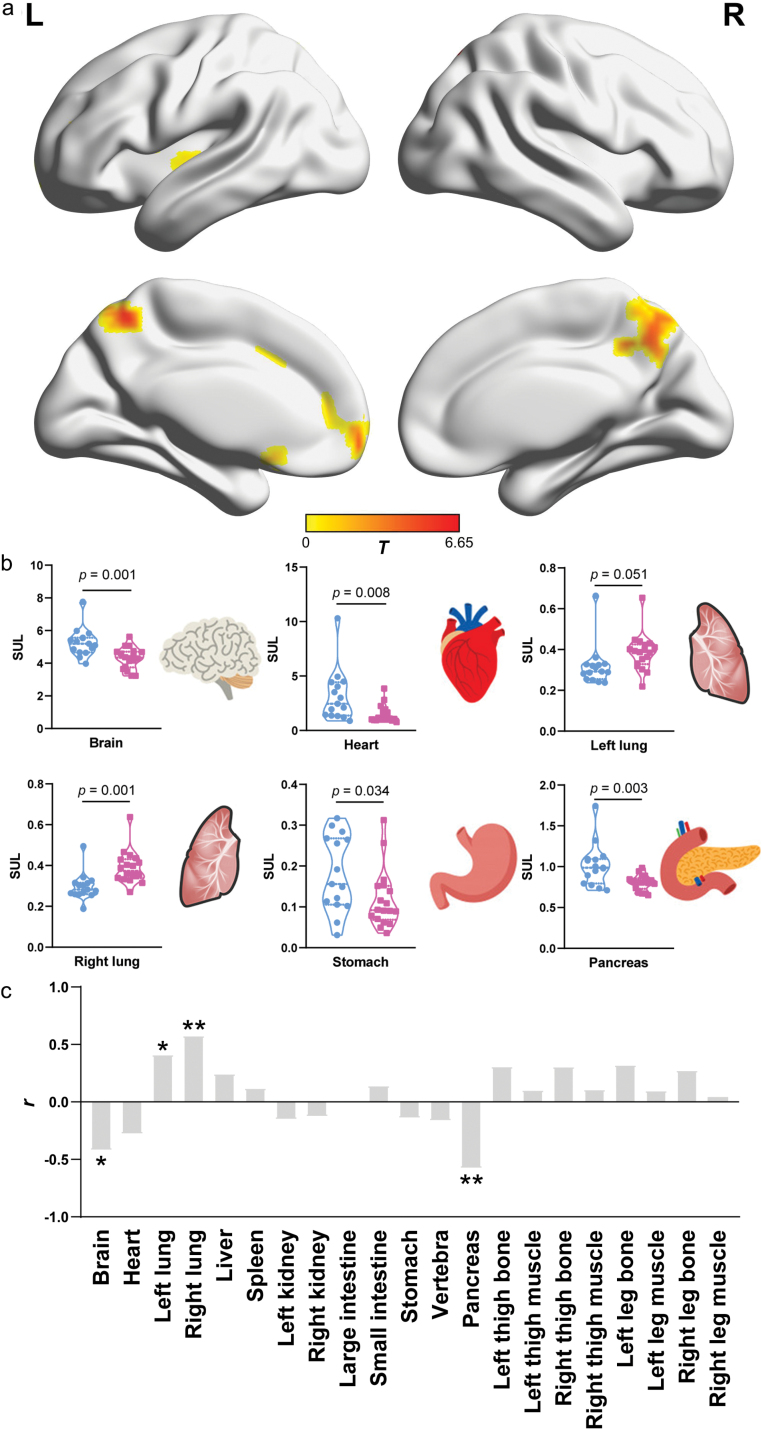
Differences in glucose uptake of major organs and parts across the human body between healthy-weight and overweight subjects. (a) T-maps indicating differences in brain SUL between healthy-weight and overweight subjects (Gaussian corrected at voxel level *P* < 0.01, cluster level *P* < 0.05). The corresponding permutation maps are shown in Supplementary Fig. S7. (b) Violin plot demonstrating differences in the mean SUL of the brain, heart, bilateral lungs, stomach, and pancreas between healthy-weight and overweight subjects. The blue points represent healthy-weight subjects, and the pink points represent overweight subjects. The error bar indicates mean ± standard deviation. (c) Associations between BMI and glucose uptake of major organs and parts across the human body among the 33 subjects. ^*^ means *P* < 0.05, ^**^ means *P* < 0.001.

Lastly, we treated the glucose uptake profiles from the 15 healthy-weight subjects as the reference standard, and compared glucose uptake between the healthy-weight and the overweight groups (Fig. 5, and the raw PET images for the 18 overweight subjects are displayed in Supplementary Fig. S6).

Compared with the healthy-weight subjects, the overweight subjects exhibited lower SULs in the bilateral precuneus and left putamen (Fig. 5a, Supplementary Fig. S7, and Supplementary Table S5). In addition, the healthy-weight and overweight groups differed in glucose uptake of the brain, heart, lung, stomach, and pancreas (Fig. 5b). Specifically, the overweight subjects demonstrated increased glucose uptake in the bilateral lungs, and decreased glucose uptake in the brain, heart, stomach, and pancreas (Fig. 5b).

We further evaluated the associations between BMI and glucose uptake in major organs and body parts. SULs of the bilateral lungs showed positive associations with BMI, but SULs in most of the brain regions and pancreas showed negative associations with BMI (Fig. 5c, Supplementary Fig. S8−S10, and Table S6).

## Discussion

Changes in metabolism contribute to the development and progression of various disorders and render them vulnerable to interventions [21]. However, research of human metabolism at the whole-body scale is still in its infancy due to technical challenges in detecting and quantifying small molecules [21–24]. In this study, using the uEXPLORER total-body PET/CT scanner, we generated the first atlas of glucose uptake across the entire human body and the zonation of glucose uptake in the brain. Since aging, cancer, and other health conditions such as obesity, metabolic syndrome, and metabolic encephalopathy give rise to altered glucose metabolism [21, 25, 26], the current results can be used as a standard or baseline for glucose uptake in the clinical treatment of these disorders. In addition, via this glucose uptake atlas, we revealed the association between glucose uptake and age, as well as the differences in glucose uptake between sexes, between the left and right sides of the body, and between the healthy-weight and overweight subjects, which in turn demonstrated the role of this atlas in understanding glucose metabolism across the human body.

Although there are several studies making endeavor to generate the total-body metabolic profiles via PET imaging [27, 28], and there are many studies describing metabolic profiles for several organs across the human body between healthy and diseased subjects [13–15], as existing PET scanners have inadequate axial FOV, the measurement of glucose uptake in major organs and parts across the human body can only be achieved via multi-bed position scan (not at the same time) [23, 24]. Thus, such atlases may fail to reveal the simultaneous and exact metabolic profiles across the entire human body. The recent total-body PET/CT system by the EXPLORER consortium, with a 194-cm-long FOV, enables the covering of the whole body and allows simultaneous tracing of biochemical processes in the entire human body [23, 24]. With the aid of this PET/CT scanner, it is possible to obtain glucose uptake images of the entire human body simultaneously and precisely.

Enabled by the uEXPLORER total-body PET/CT system, we developed a glucose uptake atlas of the human body for the first time. A recent proposal has called for an integrated human metabolic model at the system level, which would provide a scaffold for the integrative analysis of multidimensional omics data [29]. The current atlas with glucose uptake profiles across the human body may provide a platform for the investigation of metabolomics at the system level. What’s more, many diseased conditions, including obesity, diabetes, cancers, etc., involve the dysregulated metabolic activities not only in a few nearby organs, but many organs and parts across the entire body [5]. Therefore, the current atlas may also provide a useful tool and reference for the investigation of these diseased conditions involving dysregulated metabolism. In addition, the current atlas with glucose uptake profiles may enhance the understanding of glucose uptake and metabolism across the human body, demonstrated by the present investigations between glucose uptake, age, sex, lateralization, and obesity.

During the human aging process, metabolic alterations accumulate over time [16]. Generally, older age is characterized by inflammation, oxidative stress, and reduced physiological function [17]. In this study, glucose uptake of the stomach exhibited a significant association with age. A previous study has revealed the 18F-fluorodeoxyglucose (18F-FDG) uptake pattern in the stomach [30]. During human aging, histological, and physiological alterations occur in the stomach, which are mainly due to chronic insults such as helicobacter pylori and polymedication, and can be captured by the PET/CT [31, 32]. Therefore, the current association may reflect histological and physiological alterations in the stomach during aging. In the meanwhile, metabolic alterations with aging were manifested as increased SUL in the bilateral bones. Bone formation and remodeling occurs throughout the lifespan, and increasing evidences have linked bone cell activity to global energy homeostasis [33]. In fact, bone turnover and metabolic indicators are related to age and gender [34]. A previous review reports that in some cases, properties of bones improve in function, and in others, deteriorate in function with aging [35], which was in line with the current results. However, glucose uptake of most other organs and body parts exhibited inverted U-shape associations with age showing a turning point around 40−59 years. The turning point can be explained by the secondary influences of body fat and physical fitness [36, 37].

There are differences in lifestyle, body composition, hormonal secretion, and metabolic enzymes between males and females, suggesting a potential effect of sex on total-body glucose metabolism [19]. Indeed, sexual dimorphism in metabolic profiles has been observed [20, 38]. The current study revealed dimorphism of glucose metabolism in the frontal lobe of the brain. The frontal lobe is one of the most sexually dimorphic regions of the human brain, since previous studies have found differences between sexes of neuroglial relationships and brain structures in the frontal fields [39, 40]. In addition, hormones play a certain role in the metabolism of men and women. Sex hormones influence the development of female- and male-specific traits and primarily affect the structure and function of sex-specific brain regions and organs [41]. Other organs and body parts, including kidneys, showed dimorphism of glucose metabolism to some extent, which may be related to the expression and role of sex-regulating hormones in the apical and basolateral membranes of nephron epithelial cells, and the differences in structure and size under physiological conditions [41–43].

The human brain is asymmetric in both structure and function, allowing some neural functions or cognitive processes to be carried out unilaterally in specialized regions [44, 45]. In the present study, several brain regions displayed hemispheric lateralization in terms of glucose metabolism. Functional lateralization of the frontal gyrus, cingulate gyrus, globus pallidus, and temporal gyrus has been observed in both healthy subjects and diseased subjects [46–49]. As suggested by previous studies, the left–right differences in glucose metabolism in these brain regions may reflect functional lateralization of these regions. In addition, several brain regions displayed higher glucose metabolism on the left side than on the right, while other regions displayed the opposite patterns, which might reflect a compensatory mechanism in brain energy metabolism [50]. In the present study, a metabolic difference was also observed between the left and right kidneys, which may be resulted from the differences in position and size between the left and right kidneys [51].

Obesity is a complex condition mainly resulting from unbalanced energy homeostasis [52], and appears to be associated with alterations in brain structure and function [53]. Previous studies have revealed that brain regions including the putamen and the caudate nucleus are responsible for food reward processing [54]. The precuneus is involved in a variety of complex functions, and previous research has revealed the role of the precuneus in cognitive control in obesity and obesity prevention [55]. As for heart, obesity can cause changes in cardiac metabolism, which make ATP production and utilization less efficient [56]. The brain and the digestive organs closely interact in regulating food intake. However, in obesity, the balanced interaction is altered, which may lead to the altered activity in the digestive organs including the stomach [57]. In addition, the association among pancreatic fat, obesity, and metabolic disease is well-established, as obesity can result in abnormal function of the endocrine pancreas, which further leads to impaired glucose tolerance [58]. The current results demonstrated the effects of overweight on glucose metabolism in several organs including the brain, bilateral lungs, heart, and pancreas, which were well-consistent with the previous findings.

There are several limitations in the present study. First, the number of subjects were small, which is due to the advent development of the uEXPLORER scanner. We have set strict criteria for healthy subjects according to their BMI, blood glucose concentration, etc., as many studies did not exclude healthy subjects according to their BMI and blood glucose concentration. In addition, the high cost for the PET/CT also limited the number of healthy subjects. Second, partial volume effect (PVE) is not addressed. Admittedly, PVE is a huge concerning for PET analysis. However, PVE is more serious regarding to small objects, and in this study, we have focused on major organs and parts across the entire human body, which might be less affected by PVE. In addition, as the uEXPLORER has the vantages of high sensitivity, which makes the PET images less affected by PVE. Lastly, we have not collected the information including smoking and drinking due to the retrospective nature of the study. Future studies will pay more attention to the clinical information.

In summary, we developed the first glucose uptake atlas with the aid of the uEXPLORER PET/CT scanner. This atlas provides baseline glucose uptake profiles in major parts throughout the human body and variations in glucose uptake among brain regions. In addition, we demonstrated the effects of age, sex, laterality, and obesity on total-body metabolism via this atlas, which proved its role in the understanding of human metabolism and metabolomics. This glucose uptake atlas would also open a new path to the clinical diagnosis and treatment of cancers, obesity, diabetes, and other health conditions involving dysregulated metabolism.

## Materials and methods

### Participants and basic description

We collected participants with the following inclusion criteria: (i) an age between 20 and 80 years, (ii) right-handedness, (iii) 2-h postprandial blood glucose concentration in the normal range (3.9−7.8 mmol/L), and (iv) for premenopausal female subjects, they are not in their menstrual period. The exclusion criteria were as follows: (i) hypermetabolic lesions such as tumors, (ii) metabolic diseases such as diabetes, (iii) vascular diseases, and (iv) self-reported history of major or unstable medical illness. Thereafter, subjects were categorized according to body mass index (BMI), which was calculated as weight in kilograms divided by height in meters squared. BMI categories were defined as follows [38]: healthy-weight, <23.9 kg/m^2^; overweight, ≥24 kg/m^2^. Ultimately, 15 healthy-weight subjects and 18 overweight subjects were included in this study. Table 1 presents the demographic details of the 33 subjects.

**Table 1:** Demographic features of the 33 subjects.

	Healthy-weight (*n* = 15)	Overweight (*n* = 18)
Age	52.07 ± 14.23 (31−73)	55.89 ± 8.53 (42−77)
Gender	5M/10F	13M/5F
Body mass (kg)	57.27 ± 7.84 (45−70)	79.33 ± 10.64 (59−100)
Height (m)	1.66 ± 0.06 (1.55−1.73)	1.70 ± 0.08 (1.56−1.80)
BMI (kg/m^2^)	20.76 ± 2.15 (16.73−23.94)	27.33 ± 1.69 (24.21−30.86)
2 h postprandial blood glucose concentration (mmol/L)	5.52 ± 0.71 (4.4−7.2)	6.27 ± 0.83 (5.2−7.6)

^*^Continuous variables are represented as mean ± standard deviation (minimum–maximum).

### PET/CT acquisition

Prior to image acquisition, daily and weekly quality control and calibration steps were performed to ensure the quality of the PET/CT scan (details are given in the Supplementary text). The subjects received an injection of 18F-FDG via a vein near the ankle according to body mass [2.96 MBq/kg (0.08 mCi/kg)] after fasting for at least 6 h. The total-body PET/CT imaging was collected using the uEXPLORER system (United Imaging Healthcare, Shanghai, China) 50 min after the injection. The subjects are told to empty their bladder before the scan. The static total-body PET scan was performed via uEXPLORER for 600 s. The PET images were reconstructed using all 600 s of data with time of flight and point spread function modeling, 2 iterations, 20 subsets, a matrix size of 192 × 192, a slice thickness of 2.89 mm, a pixel size of 3.125 × 3.125 × 2.886 mm^3^ with a Gaussian filter (full width at half maximum = 3 mm), and all necessary corrections (including attenuation and scatter correction).

### Glucose uptake mapping

Major organs and parts across the entire human body, including the brain, heart, bilateral lungs, vertebrae, stomach, liver, spleen, pancreas, large intestine, small intestine, bilateral kidneys, bilateral thigh bones, bilateral thigh muscles, biliteral calf bones, and bilateral calf muscles, were annotated by a certified radiologist using the active contour tool ITK-SNAP (version 3.6.0) (Fig. 1). Thereafter, the annotated organs and body parts were inspected by another certified radiologist, and relevant corrections were performed. At this stage, masks of these organs and body parts were obtained. The mean SUVs normalized SUL of 18F-FDG in these organs and body parts was calculated. First, SUV was calculated as the ratio of radioactivity concentration divided by the administered dose at the time of injection divided by body weight. Lean body mass was then calculated according to Hume [59] (Supplementary Equation). The SUV value was then normalized by lean body mass to obtain the SUL value.

In addition, brain PET images were extracted from the total-body PET images. Thereafter, statistical parametric mapping software (SPM8) was used for the processing of brain PET images. Specifically, brain PET images were spatially registered to the Montreal Neurologic Institute template, and an SUL was calculated for each voxel within the brain. Then, the SUL map was smoothed using a 6-mm full width at half maximum Gaussian isotropic kernel.

### Analysis of associations among different parameters

The association between age and glucose uptake was evaluated by performing Pearson correlation or quadratic regression analysis between age and the mean SUL in major organs and parts throughout the human body in the healthy-weight group. The best-fitting model was determined using the Akaike information criterion (AIC). The model with a smaller AIC value was used to describe the association between glucose uptake and age. The association between age and brain glucose uptake was evaluated by performing Pearson correlation analysis between age and brain SUL maps.

In order to investigate the total-body differences in glucose uptake between men and women in healthy-weight group, an independent *t*-test was used to compare the mean SULs of major organs and body parts throughout the human body between sexes. General linear model was used to compare brain SUL ratio maps (standardized by z-score approach) between male and female subjects, with age as a nuisance covariate.

In terms of glucose uptake differences between the left and right sides of the body, paired *t*-test was applied to compare the mean SULs of the bilateral lungs, kidneys, muscles and bones of the thighs and calves, and bilateral brain subregions (each side of brain subregions correspond to the contralateral body parts) according to brain parcellation by the automated anatomical labeling atlas.

Lastly, differences of glucose uptake across the whole body between healthy-weight and overweight groups were evaluated. An independent *t*-test was used to compare the differences in the mean SULs of major organs and body parts throughout the human body between the two groups. General linear model was used to compare brain SUL ratio maps (standardized by z-score approach) between healthy-weight and overweight subjects, with age and gender as nuisance covariates.

## Supplementary Material

loac030_suppl_Supplementary_Material

## Data Availability

Data will be made available on reasonable request.
