# Supplementary material for: An atlas of glucose uptake across the entire human body as measured by the total-body PET/CT scanner: a pilot study
Source: Life Metab. 2022 Oct 27;1(2):190–9. doi: 10.1093/lifemeta/loac030 (PMC11749875; doi:10.1093/lifemeta/loac030)
Supplement: loac030_suppl_Supplementary_Material [file loac030_suppl_Supplementary_Material.pdf]

Quality control and calibration of the uEXPLORER PET/CT system

Quality control and calibration steps performed to the uEXPLORER PET/CT system consist of two major types, passive quality control and active quality control.

Passive quality control

Passive quality control is performed daily, which consists of quality checks and calibration of the count rate, voltage, data links. Calibrations depends on the temperature and humidity are also performed. The purpose is to confirm the status of the scan before use to ensure that it can meet the requirement of clinical use and that the images meet the diagnostic needs.

Active quality control

Active quality control is performed weekly, with the help of a cylindrical water phantom. The phantom was a cylinder filled with water and approximately 1 mCi 18F-FDG. Through active quality control, we can calibrate energy drift, time-of-flight module, coincidence map, uniformity, etc.

The calculation of lean body mass proposed by Hume:

$$\mathrm{LBM(M)} = 0.32810 \times \mathrm{weight} + 0.33929 \times \mathrm{height} - 29.5336 \quad (1)$$

$$\mathrm{LBM(W)} = 0.29569 \times \mathrm{weight} + 0.41813 \times \mathrm{height} - 43.2933 \quad (2)$$

The unit of weight is kilogram, the unit of height is centimeter.

LBM: lean body mass, M: male, W: women.

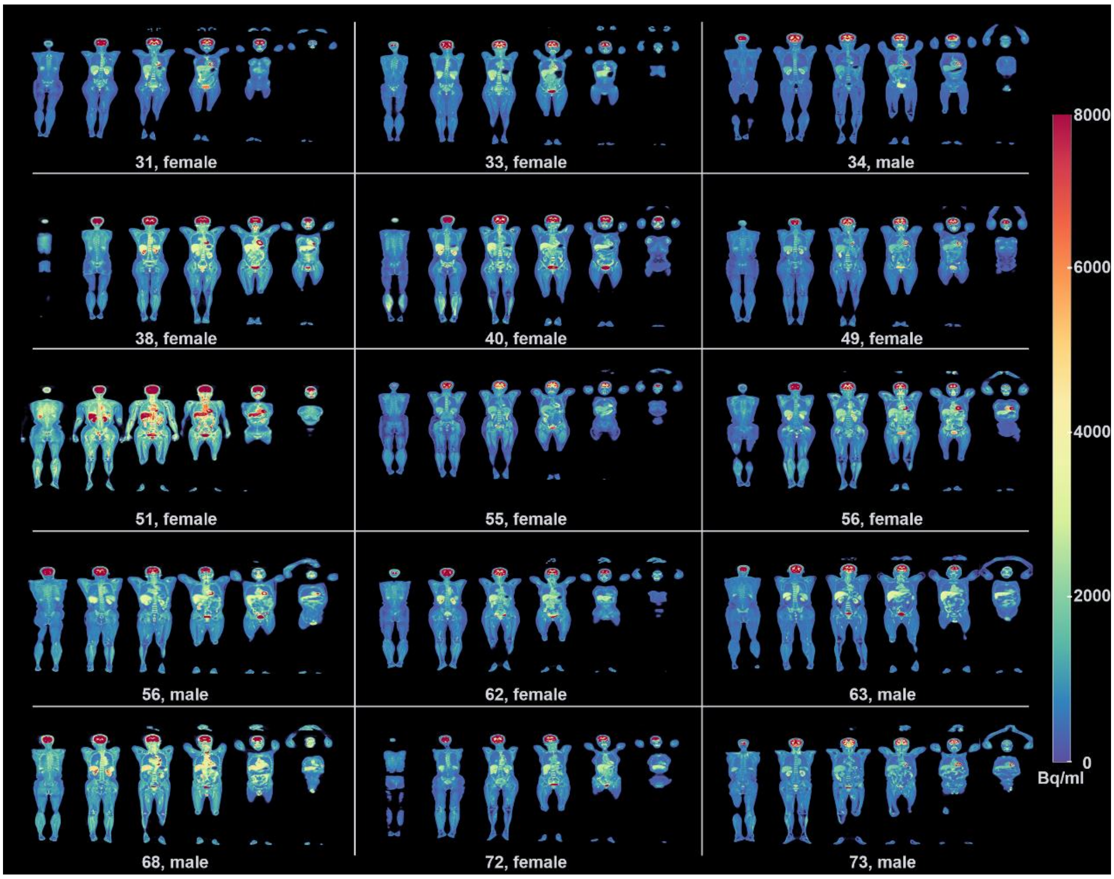

Supplementary Figure S1. Visualization of raw PET images for the 15 healthy subjects. The unit of each voxel value is Bq/ml. We choose to display 6 coronal slices for each subject. However, due to different body sizes and body position, some of the slices only show parts of the body.

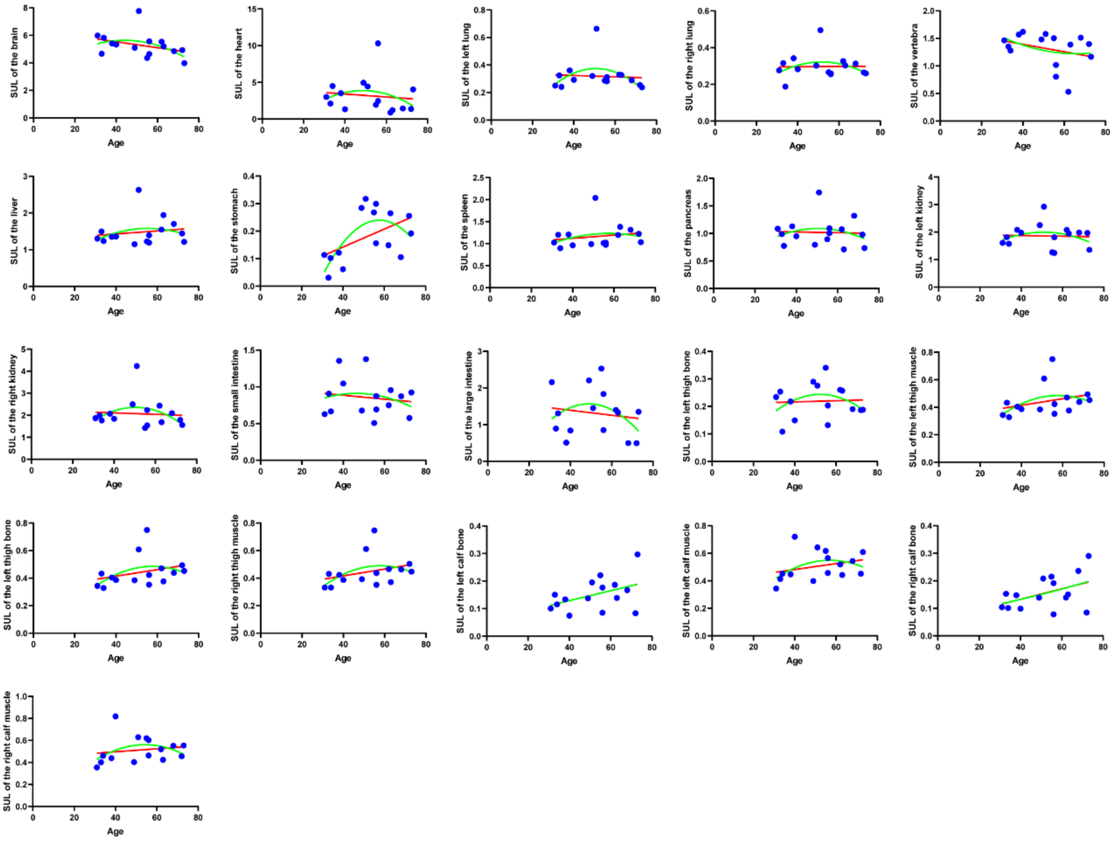

Supplementary Figure S2. Associations between age and mean SULs of major organs and body parts throughout the body in the healthy weight subjects. Scatter plots showing the association of age with the SULs of the brain, heart, left lung, right lung, vertebra, liver, stomach, spleen, pancreas, left kidney, right kidney, small intestine, large intestine, left thigh bone, left thigh muscle, right thigh bone, right thigh muscle, left calf bone, left calf muscle, right calf bone, right calf muscle. The red line represents the best fitting line according to linear regression, and the green curve represents the quadratic regression curve.

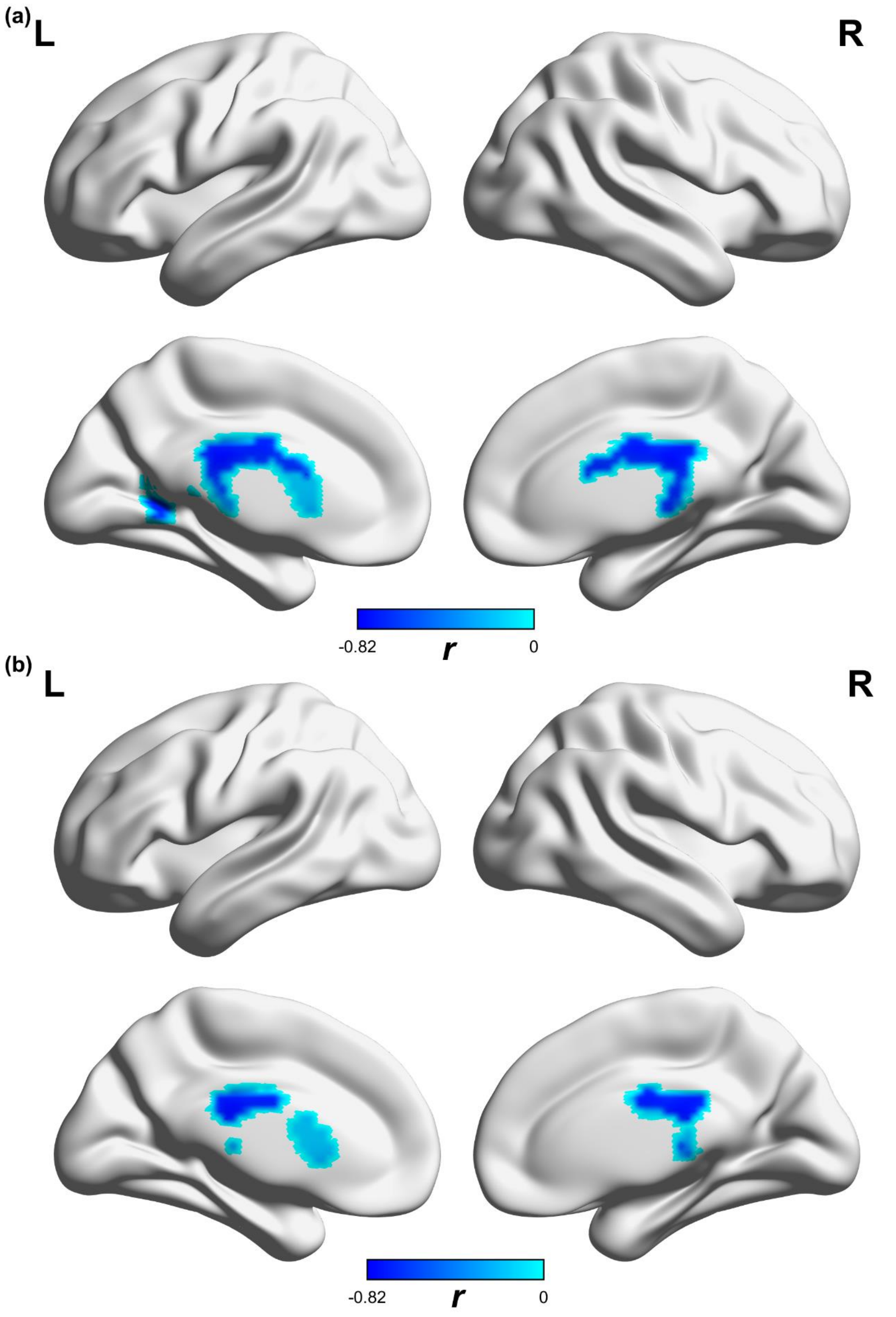

Supplementary Figure S3. Correlation maps between brain SUL and age shown significantly negative correlations between SUL of the corpus callosum and age in the 15 healthy weight subjects. (a) Significant correlations are defined as Pearson correlation analysis at $p < 0.05$, with Gaussian random

field correction at voxel level $p < 0.01$ and cluster level $p < 0.05$. (b) Significant correlations are defined as permutation test (1000 times) with $p < 0.01$. The permutation test is conducted in this way: Via the Pearson correlation analysis between ages and brain SUL maps, the original *r* value is obtained. Then the ages of the 15 healthy weight subjects are rearranged randomly for 1000 times. In each permutation, Pearson's correlation analysis is performed between the rearranged ages and brain SUL maps, and a coefficient value *r* is obtained. After 1000 times permutation test, we obtained 1000 r values. The p value is calculated as the ratio of absolute permutation-obtained *r* values larger than the original *r* value.

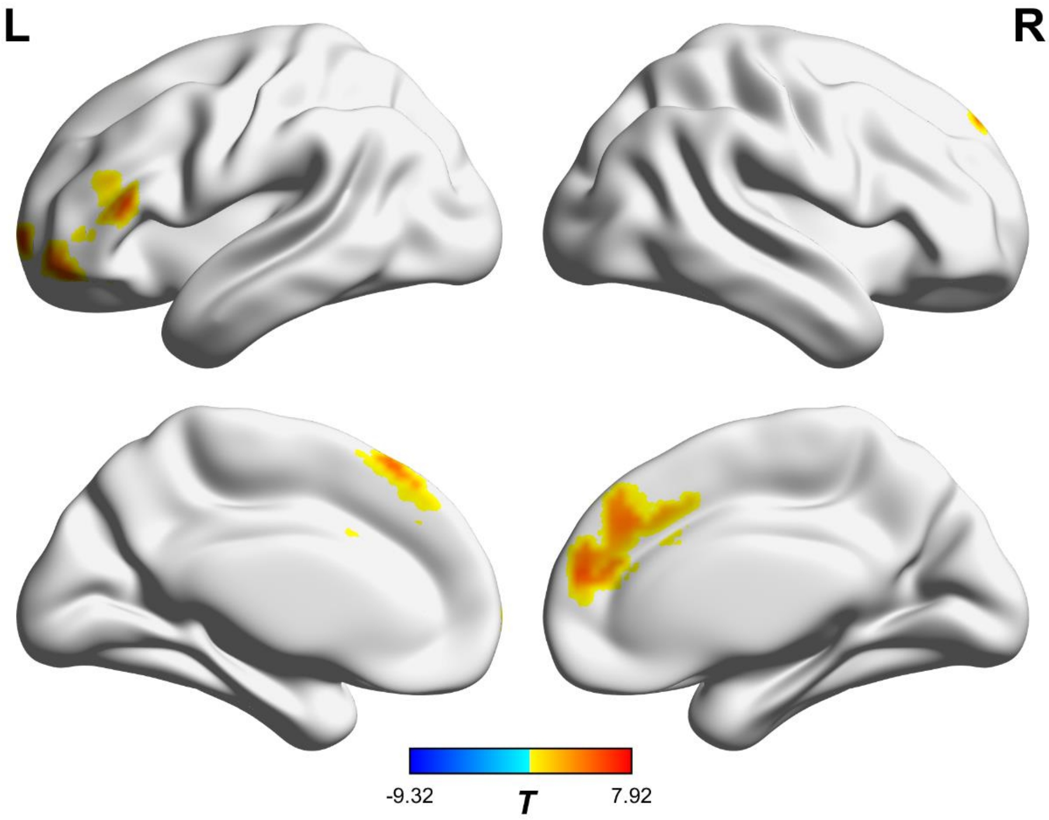

Supplementary Figure S4. Comparisons of SUL ratio maps of the brain between male and female subjects with permutation test (1000 times) at $p < 0.05$ and threshold-free cluster enhancement to control false positives. The permutation test is conducted in this way: Via T-test between SUL maps of male and female subjects, the original T statist is obtained. Then the 15 healthy weight subjects are rearranged randomly into two groups ($n_1=5$, $n_2=10$) for 1000 times. In each permutation, t-test is performed between the SUL maps of the two groups, and a T value is obtained. After 1000 times permutation test, we obtained 1000 T values. The p value is calculated as the ratio of permutation-obtained T values larger than the original T value.

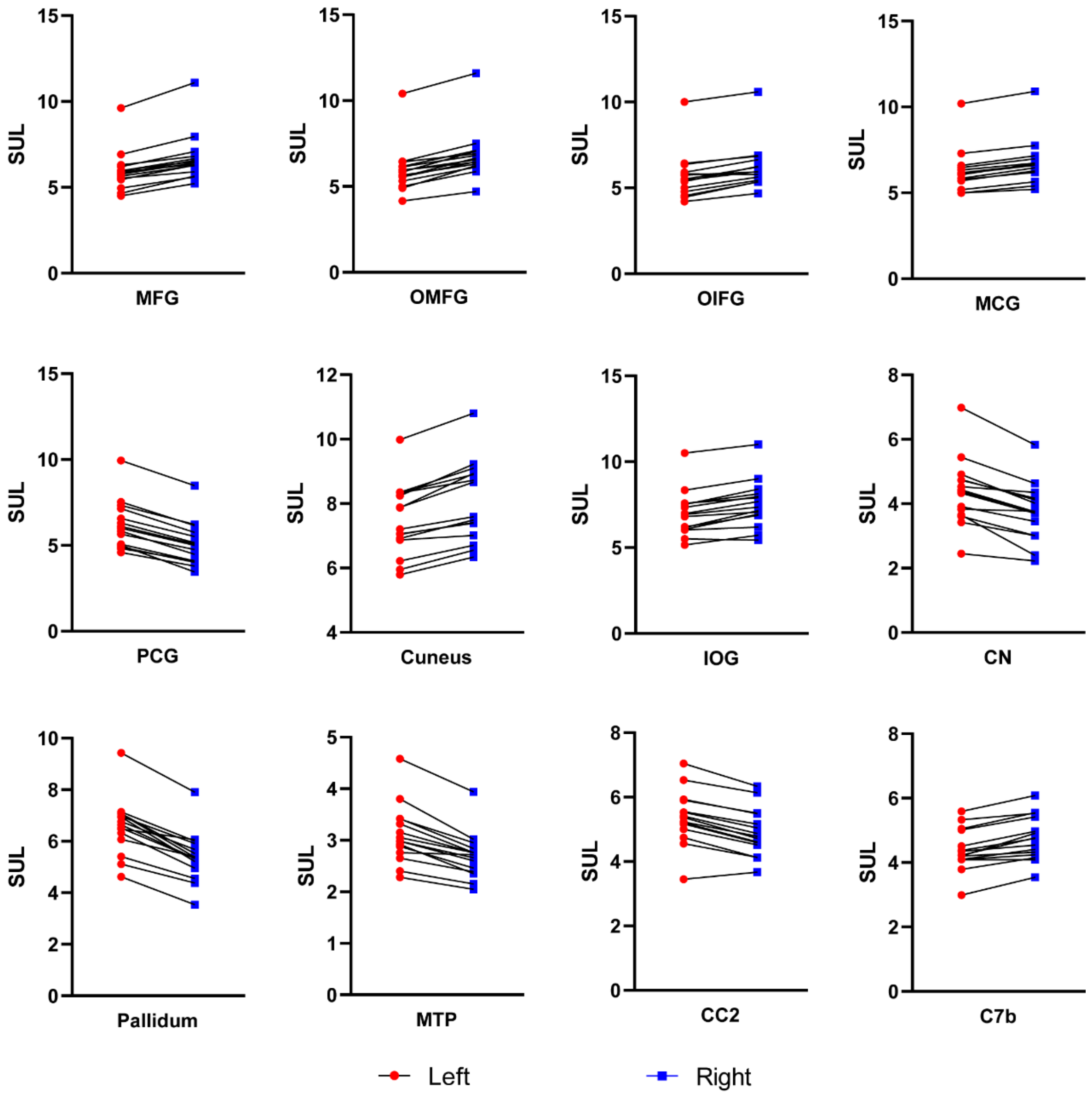

Supplementary Figure S5. Paired t-test results of SULs in several brain regions between the left and right sides of the body in the healthy weight subjects. The t-statistics and p-value are demonstrated in Supplementary Table S4.

Abbreviations: Abbreviations: MFG: middle frontal gyrus; OMFG: orbital part of middle frontal gyrus; OIFG: opercular part of inferior frontal gyrus; MCG: middle cingulate gyrus; PCG: posterior cingulate gyrus; IOG: inferior occipital gyrus; CN: caudate nucleus; MTP: middle temporal pole; CC2: crus II of the cerebellum; C7b: cerebellum 7b.

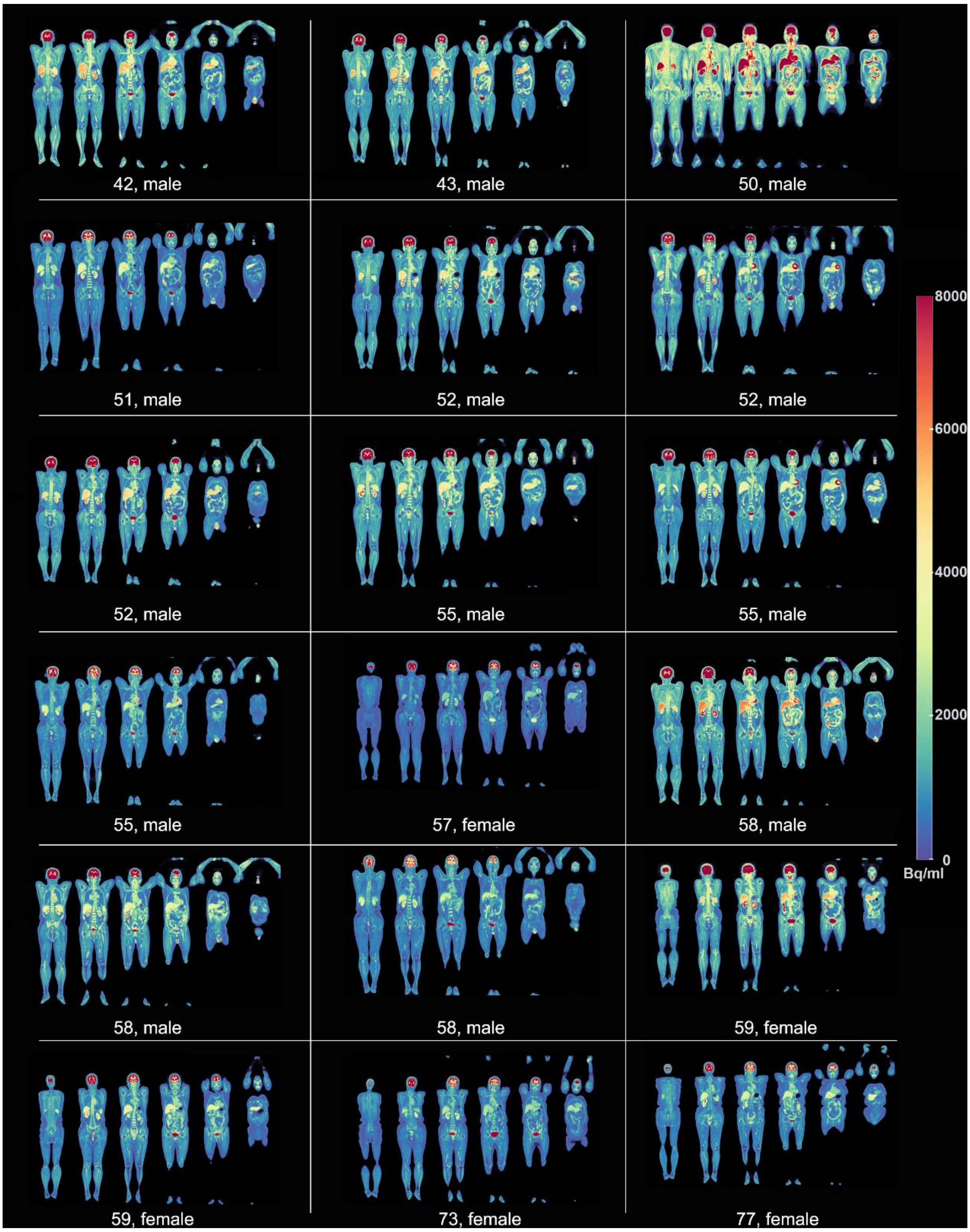

Supplementary Figure S6. Visualization of raw PET images for the 18 overweight subjects. The unit of each voxel value is Bq/ml. We choose to display 6 coronal slices for each subject.

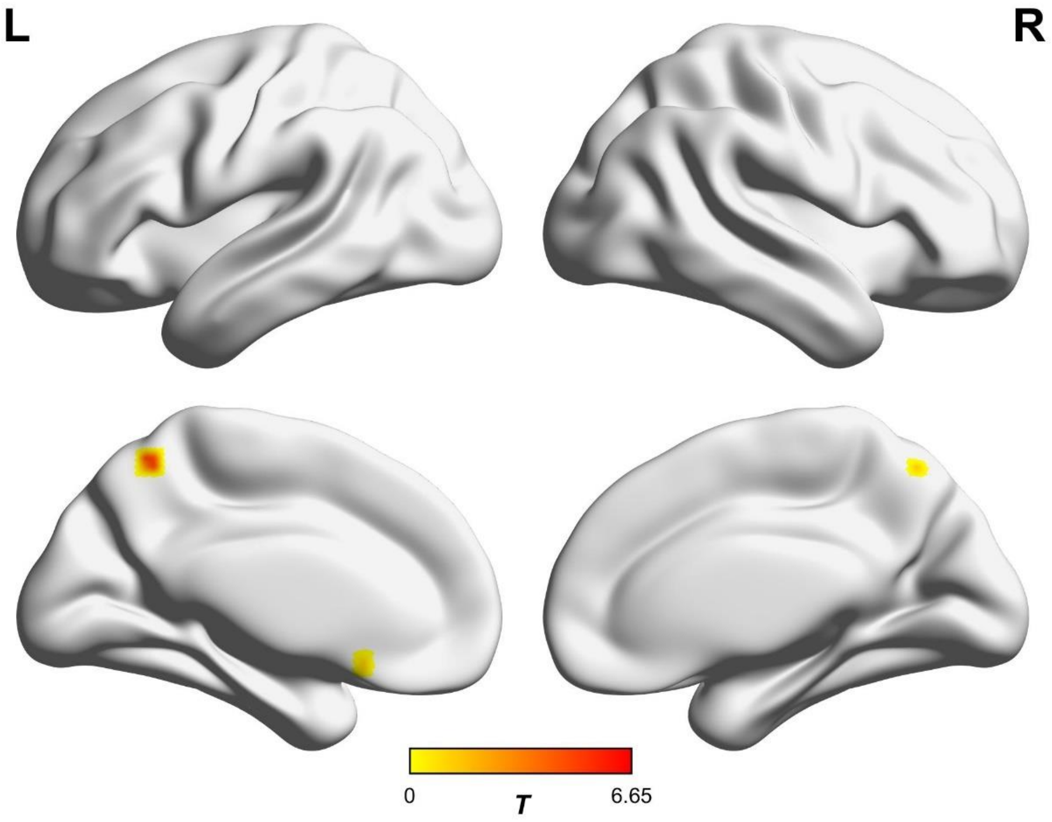

Supplementary Figure S7. Comparisons of SUL ratio maps of the brain healthy weight and overweight subjects with permutation test (1000 times) at $p < 0.05$ and threshold-free cluster enhancement to control false positives. The permutation test is conducted in this way: Via the T-test between SUL maps of healthy weight and overweight subjects, the original T statist is obtained. Then the 33 subjects are rearranged randomly into two groups (n_1=15, n_2=18) for 1000 times. In each permutation, t-test is performed between the SUL maps of the two groups, and a T value is obtained. After 1000 times permutation test, we obtained 1000 T values. The p value is calculated as the ratio of permutation-obtained T values larger than the original T value.

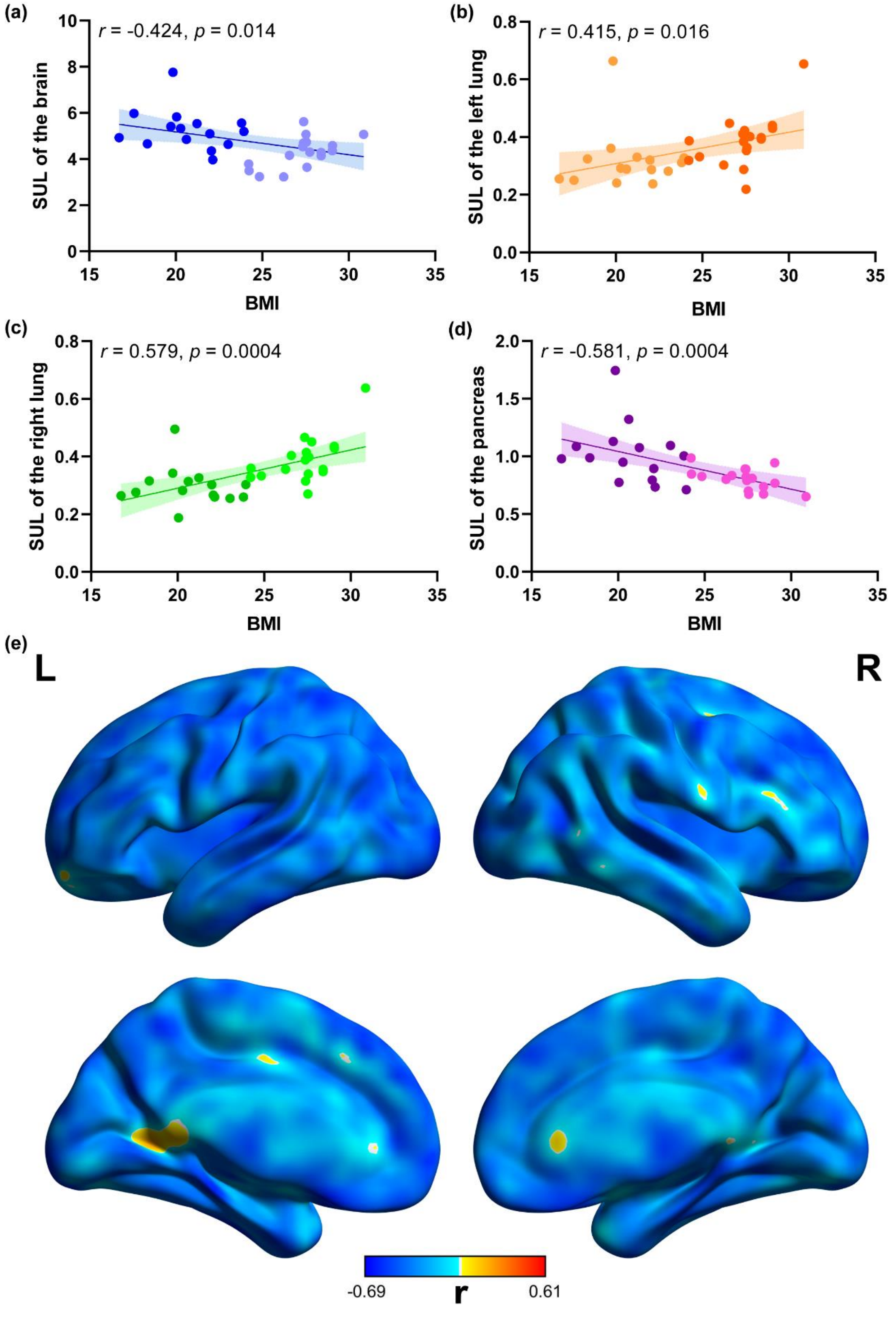

Supplementary Figure S8. Associations between BMI and glucose uptake of major organs and parts across the human body among the enrolled 33 subjects. (a) Scatter plot of the association between BMI and mean SUL of the (a) brain, (b) left lung, (c) right lung, (d) pancreas, and (e) voxels across the entire brain. For Figure S8(a)-(d), the correlation results after outlier removal are shown in Supplementary

Figure S9. Significant correlations between BMI and brain regions are visualized in Supplementary Figure S10. The filled area indicates the 95% confidence interval, healthy weight and overweight subjects are distinguished by different colors in the scatter plot.

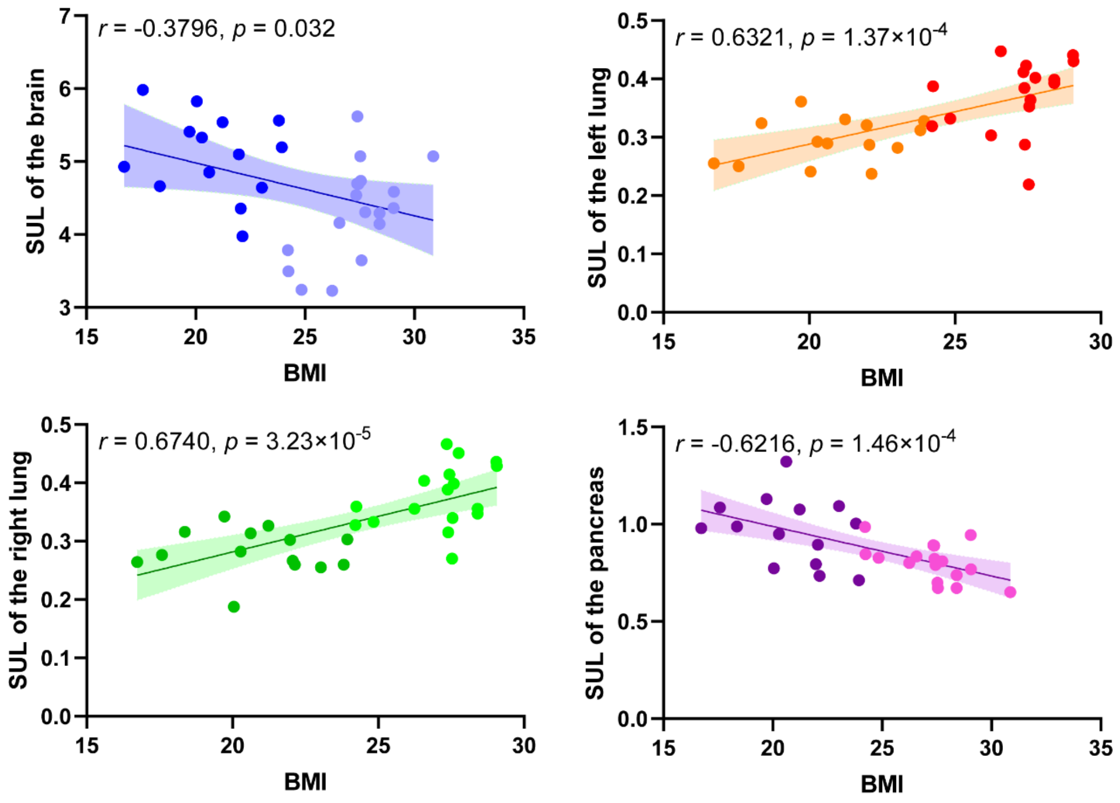

Supplementary Figure S9. Associations between BMI and glucose uptake of major organs and parts across the human body among the enrolled subjects after outlier removal. Scatter plot of the association between BMI and mean SUL of the (a) brain, (b) left lung, (c) right lung, (d) pancreas. The filled area indicates the 95% confidence interval, healthy weight and overweight subjects are distinguished by different colors in the scatter plot.

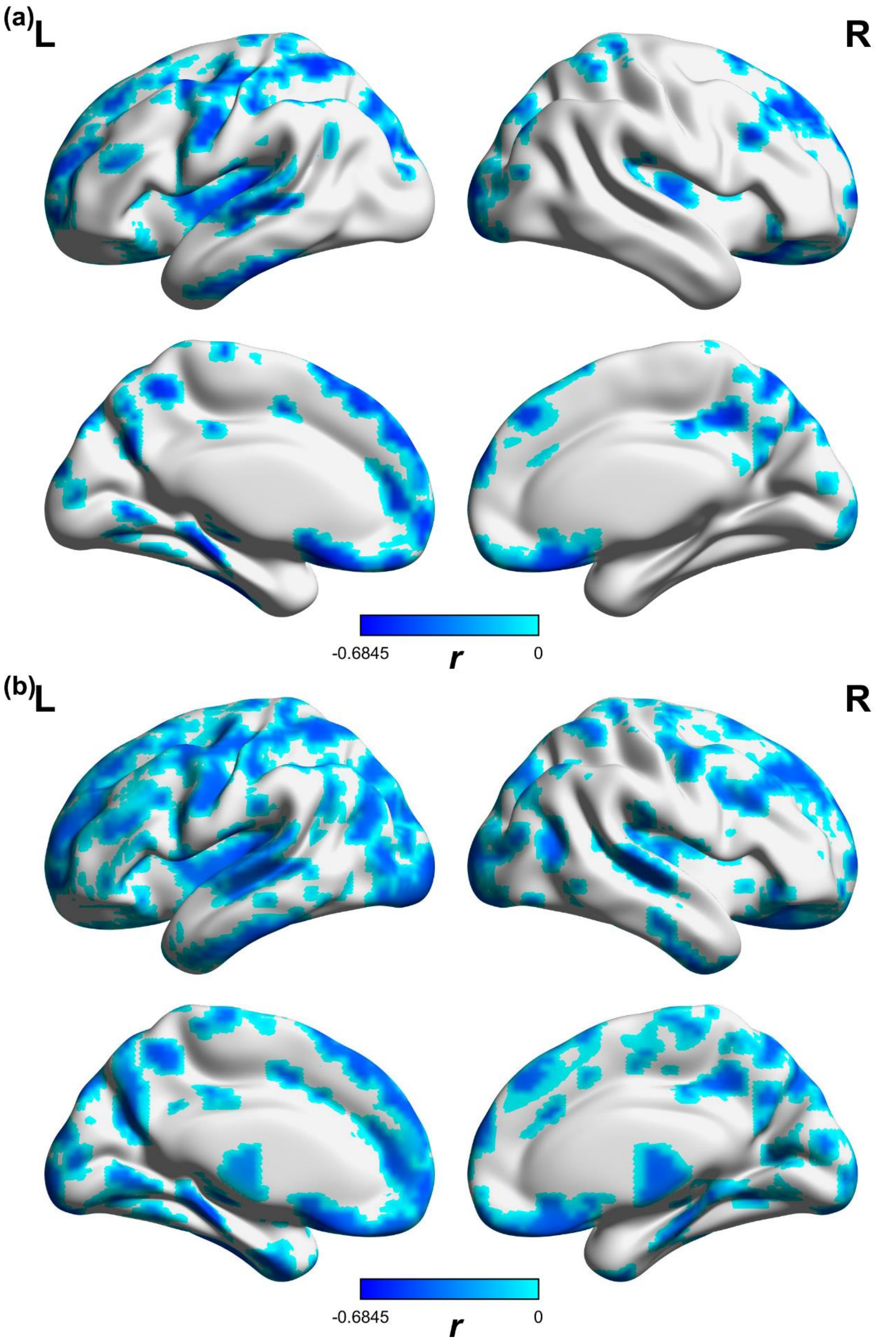

Supplementary Figure S10. Significant associations between BMI and glucose uptake of the brain among the enrolled 33 subjects. (a) Significant correlations are defined as Pearson correlation analysis at $p < 0.05$, with Gaussian random field correction at voxel level $p < 0.01$ and cluster level $p < 0.05$. (b)

Significant correlations are defined as permutation test (1000 times) with $p < 0.01$. The permutation test is conducted in this way: Via the Pearson correlation analysis between BMIs and brain SUL maps, the original *r* value is obtained. Then the BMIs of the 33 subjects are rearranged randomly for 1000 times. In each permutation, Pearson's correlation analysis is performed between the rearranged BMIs and brain SUL maps, and a coefficient value *r* is obtained. After 1000 times permutation test, we obtained 1000 r values. The p value is calculated as the ratio of absolute permutation-obtained *r* values larger than the original *r* value.

Supplementary Table S1. Associations between age and mean SUL of major organs and body parts across the entire body by linear regression and quadratic regression in the healthy weight subjects.

Body parts	Linear regression				Quadratic regression			
	r^2	$p_{\_reg}$*	$p_{_perm}$ǂ	AIC	r^2	$p_{\_reg}$*	$p_{_perm}$ǂ	AIC
Brain	0.1212	0.20	0.22	2.65	0.1974	0.27	0.27	2.69
Heart	0.0146	0.67	0.71	4.76	0.0765	0.62	0.65	4.83
Left lung	0.0047	0.81	0.82	-1.53	0.2376	0.20	0.18	-1.66
Right lung	0.0001	0.97	0.98	-2.38	0.1137	0.48	0.50	-2.37
Vertebra	0.0956	0.26	0.26	0.60	0.1115	0.49	0.50	0.72
Liver	0.0231	0.59	0.63	1.09	0.0730	0.63	0.68	1.17
Stomach	0.2479	0.06	0.06	-1.99	0.4748	0.02	0.02	-2.22
Spleen	0.0344	0.51	0.56	0.46	0.0681	0.65	0.69	0.55
Pancreas	0.0015	0.89	0.90	0.35	0.0593	0.69	0.72	0.42
Left kidney	0.0015	0.89	0.90	1.34	0.0817	0.60	0.60	1.39
Right kidney	0.0048	0.81	0.85	2.25	0.1472	0.38	0.40	2.23
Small intestine	0.0215	0.60	0.62	0.31	0.0501	0.73	0.74	0.41
Large intestine	0.0221	0.60	0.60	2.15	0.1320	0.43	0.43	2.16
Left thigh bone	0.0024	0.86	0.86	-2.50	0.1247	0.45	0.44	-2.50
Left thigh muscle	0.0948	0.26	0.27	-1.48	0.1792	0.31	0.30	-1.44
Right thigh bone	0.0009	0.92	0.91	-2.67	0.1801	0.30	0.31	-2.73
Right thigh muscle	0.1043	0.24	0.26	-1.49	0.1953	0.27	0.27	-1.46
Left calf bone	0.1851	0.11	0.10	-2.81	0.1851	0.29	0.29	-2.68
Left calf muscle	0.0844	0.29	0.29	-1.56	0.1792	0.31	0.32	-1.53
Right calf bone	0.1952	0.10	0.11	-2.77	0.1955	0.27	0.27	-2.63
Right calf muscle	0.0266	0.56	0.61	-1.24	0.1486	0.38	0.38	-1.24

* The $p_{\_reg}$ are p values from the regression analysis. $P_{\_reg}$ values are uncorrected. The corrected p values will be calculated as uncorrected $p_{\_reg}$ values times 21 for Bonferroni correction.

ǂ The $p_{\_perm}$ values are p values calculated via 1000-time permutation test.

Supplementary Table S2. SUL of brain regions with significant associations with age (Permutation test with $p < 0.01$ was considered statistically significant).

Brain regions	Cluster size*	MNI coordinates			*r* value‡
		x	y	z	
Corpus Callosum	533	-6	6	12	-0.8203

* represents number of voxel.

‡ means Pearson's correlation coefficient of the peak voxel within the cluster.

Abbreviations: MNI, Montreal Neurological Institute.

Supplementary Table S3. Brain regions with significant differences in SUL between female and male healthy subjects (Gaussian random field corrected at voxel level $p < 0.01$ and cluster level $p < 0.05$).

Brain regions	Cluster size*	MNI coordinates			*T* value‡	Types of difference
		x	y	z		
Left IFGT	349	-51	30	15	7.9188	Female > Male
Right SFG	432	21	24	21	-9.3161	Female < Male
Bilateral SMFG	445	6	51	18	6.6891	Female > Male

* represents number of voxel.

‡ means T value of the peak voxel within the cluster.

Abbreviations: MNI, Montreal Neurological Institute; IFGT, inferior frontal gyrus, triangular part; SFG, superior frontal gyrus; SMFG, medial part of the superior frontal gyrus.

Supplementary Table S4. Comparisons of the mean SULs between left and right brain regions in the healthy weight subjects.

Brain region	p value	t-statistic[#]
MFG	6.14×10^{-8}	-10.3435
OMFG	1.17×10^{-8}	-11.7917
OIFG	5.84×10^{-6}	-7.0426
MCG	1.60×10^{-10}	-16.3648
PCG	1.03×10^{-10}	16.91181
Cuneus	3.93×10^{-7}	-8.89003
IOG	3.85×10^{-6}	-7.30947
Caudate nucleus	4.66×10^{-6}	7.186301
Pallidum	1.21×10^{-8}	11.7588
MTP	8.38×10^{-7}	8.342083
CC2	7.63×10^{-7}	8.409143
C7b	$5.19\times10\text{-}^{6}$	-7.11753

* All the p values are uncorrected. The corrected p values will be calculated as uncorrected p values times 54 for Bonferroni correction since there are 54 subregions which have contralateral parts in the AAL brain atlas (116 total subregions). Raw $p < 0.0009$ (0.05/54) is considered statistically significant.

[#] A positive t-statistic indicates that the mean SUL of the left side is greater than that of the right side, and vice versa.

Abbreviations: OMFG: orbital part of middle frontal gyrus; OIFG: orbital part of inferior frontal gyrus; MCG: middle cingulate gyrus; PCG: posterior cingulate gyrus; IOG: inferior occipital gyrus; MTP: middle temporal pole; CC2: crus II of the cerebellum; C7b: cerebellum 7b.

Supplementary Table S5. Brain regions with significant differences in SUL between healthy weight and overweight subjects (Gaussian random field corrected at voxel level $p < 0.01$ and cluster level $p < 0.05$).

Brain regions	Cluster size*	MNI coordinates			*T* value‡	Types of difference
		x	y	z		
Left putamen	575	-24	12	6	6.6455	Healthy weight > Overweight
Precuneus	263	-6	-57	57	5.638	Healthy weight > Overweight

* represents number of voxel.

‡ means T value of the peak voxel within the cluster.

Abbreviations: MNI, Montreal Neurological Institute.

Supplementary Table S6. Associations between age and mean SUL of major organs and body parts across the entire body by correlation analysis in the 33 enrolled subjects.

Body parts	Correlation analysis		
	r	$p_{\_reg}$*	$P_{\_perm}$ ǂ
Brain	-0.4236	0.0140	0.014
Heart	-0.2829	0.1107	0.114
Left lung	0.4147	0.0164	0.009
Right lung	0.5790	0.0004	<0.001
Vertebra	-0.1669	0.3533	0.341
Liver	0.2473	0.1651	0.152
Stomach	-0.1442	0.4233	0.429
Spleen	0.1220	0.4987	0.474
Pancreas	-0.5806	<0.0001	<0.001
Left kidney	-0.1563	0.3850	0.373
Right kidney	-0.1302	0.4703	0.480
Small intestine	0.1438	0.4246	0.453
Large intestine	6.523×10^{-5}	0.9997	0.999
Left thigh bone	0.3115	0.0777	0.059
Left thigh muscle	0.1056	0.5586	0.583
Right thigh bone	0.3094	0.0798	0.130
Right thigh muscle	0.1110	0.5385	0.812
Left calf bone	0.3239	0.0659	0.085
Left calf muscle	0.1008	0.5768	0.557
Right calf bone	0.2770	0.1187	0.069
Right calf muscle	0.0428	0.8132	0.558

* The $p_{\_reg}$ are p values from the regression analysis. $P_{\_reg}$ values are uncorrected. The corrected p values will be calculated as uncorrected $p_{\_reg}$ values times 21 for Bonferroni correction.

ǂ The $p_{\_perm}$ values are p values calculated via 1000-time permutation test.
